# Inhaled Submicron Particle Paclitaxel (NanoPac) Induces Tumor Regression and Immune Cell Infiltration in an Orthotopic Athymic Nude Rat Model of Non-Small Cell Lung Cancer

**DOI:** 10.1089/jamp.2018.1517

**Published:** 2019-10-01

**Authors:** James Verco, William Johnston, Michael Frost, Michael Baltezor, Philip J. Kuehl, Anita Lopez, Andrew Gigliotti, Steven A. Belinsky, Ronald Wolff, Gere diZerega

**Affiliations:** ^1^ US Biotest, Inc., San Luis Obispo, California.; ^2^ Western Diagnostic Services Laboratory, Santa Maria, California.; ^3^ CritiTech, Inc., Lawrence, Kansas.; ^4^ Lovelace Biomedical, Albuquerque, New Mexico; ^5^ RK Wolff—Safety Consulting, Fort Myers, Florida.; ^6^ NanOlogy, LLC, Fort Worth, Texas.

**Keywords:** aerosol, athymic rat, Calu-3, chemotherapy, inhaled, jet nebulizer, lung cancer, NanoPac, non-small cell lung cancer (NSCLC), nude rat, orthotopic, paclitaxel, particle, preclinical, rodent, submicron

## Abstract

***Background:*** This study evaluated the antineoplastic and immunostimulatory effects of inhaled (IH) submicron particle paclitaxel (NanoPac^®^) in an orthotopic non-small cell lung cancer rodent model.

***Methods:*** Male nude rats were whole body irradiated, intratracheally instilled with Calu-3 cancer cells and divided into six treatment arms (*n* = 20 each): no treatment (Group 1); intravenous nab-paclitaxel at 5.0 mg/kg once weekly for 3 weeks (Group 2); IH NanoPac at 0.5 or 1.0 mg/kg, once weekly for 4 weeks (Groups 3 and 4), or twice weekly for 4 weeks (Groups 5 and 6). Upon necropsy, left lungs were paraffin embedded, serially sectioned, and stained for histopathological examination. A subset was evaluated by immunohistochemistry (IHC), anti-pan cytokeratin staining AE1/AE3^+^ tumor cells and CD11b^+^ staining dendritic cells, natural killer lymphocytes, and macrophage immune cells (*n* = 2, Group 1; *n* = 3 each for Groups 2–6). BCL-6 staining identified B lymphocytes (*n* = 1 in Groups 1, 2, and 6).

***Results:*** All animals survived to scheduled necropsy, exhibited no adverse clinical observations due to treatment, and gained weight at the same rate throughout the study. Histopathological evaluation of Group 1 lung samples was consistent with unabated tumor growth. Group 2 exhibited regression in 10% of animals (*n* = 2/20). IH NanoPac-treated groups exhibited significantly higher tumor regression incidence per group (*n* = 11–13/20; *p* < 0.05, *χ*^2^). IHC subset analysis revealed tumor-nodule cluster separation, irregular borders between tumor and non-neoplastic tissue, and an increased density of infiltrating CD11b^+^ cells in Group 2 animals (*n* = 2/3) and in all IH NanoPac-treated animals reviewed (*n* = 3/3 per group). A single animal in Group 4 and Group 6 exhibited signs of pathological complete response at necropsy with organizing stroma and immune cells replacing areas presumed to have previously contained adenocarcinoma nodules.

***Conclusion:*** Tumor regression and immune cell infiltration were observed in all treatment groups, with an increased incidence noted in animals receiving IH submicron particle paclitaxel treatment.

## Introduction

Non-small cell lung cancer (NSCLC) therapy has seen an evolution over the past few years by aiding the host's immune system in the fight against cancer. Where NSCLC tumors often develop immunosuppressive microenvironments allowing for unchecked tumor growth,^(1)^ immunotherapy (IT), specifically checkpoint inhibitors, aims to overcome the immunosuppressive tumor microenvironment (TME) by allowing the endogenous immune system to identify and eliminate cancer cells or by enhancing the cytotoxic potential of immune effector cells in the immunogenic cell death cycle.^(2)^ While significant survival benefits have been observed in the specific NSCLC populations indicated for IT, efficacy of monotherapy is often limited due to the heterogeneous nature of the NSCLC TME^(3)^ and to the repopulation of tumor cells between treatment administrations.^(4)^

To maximize tumoricidal effects, it has been hypothesized that the immune system can be primed with systemic chemotherapy ahead of IT to reinstate or enhance immunosurveillance.^(3,5)^ The priming effect of systemic chemotherapy would provide direct tumoricidal activity releasing tumor-specific antigens, as well as eliciting drug-induced immunomodulatory effects.^(3,5–12)^ For example, paclitaxel inhibits tubulin depolymerization in the late G2/M cell cycle phase as a primary cytotoxic mechanism, and was shown to stimulate the endogenous immune system's tumoricidal capability when administered at low doses^(3,7,9,13,14)^ through increased populations of T_effector_ cells and antigen-presenting dendritic cells,^(3,6,9,15,16)^ increased interleukin (IL)-12 secretion to enhance the tumoricidal activity of T cells, natural killer (NK) cells, and for the activation of M1 macrophages.^(3,7,15,17)^ Furthermore, paclitaxel decreases the population (or inhibits the function) of immunosuppressive myeloid-derived suppressor cells^(3,8,16)^ and T_reg_ cells^(3,6,7,15,16)^ known to populate NSCLC lesions.^(1,18)^ While a combination of chemotherapy and IT has the potential to maximize efficacy, combined regimens have been associated with severe systemic adverse effects.^(19,20)^ Local administration of chemotherapy ahead of IT therefore has the potential to offer a directed immunomodulatory effect without adding toxic exposure to nontarget organs.^(21,22)^

### NanoPac^®^

NanoPac is uncoated submicronized paclitaxel processed through compressed antisolvent precipitation into a narrow size distribution and delivered in a reconstituted suspension in physiological saline.^(23–26)^ The particles have a specific surface area of 39 m^2^/g [measured by the USP BET method (United States Pharmacopeia 31, General Chapter <846> Specific Surface Area)] and poured density of 0.06 g/cm^3^. In comparison, milled paclitaxel particles of a similar dimension have a specific surface area of 10.5 m^2^/g and a poured density of 0.26 g/cm^3^. The extended residence of paclitaxel has been demonstrated clinically in tissue sampled *ex vivo* from prostates removed 30 days after single intraprostatic administration of NanoPac (NCT03077659). The same process has been used to produce submicronized docetaxel which has also demonstrated extended residence and antineoplastic activity in genitourinary xenografts implanted in nude mice.^(27)^

Inhaled (IH) paclitaxel for locoregional NSCLC treatment has demonstrated proof of principle in a variety of preclinical models.^(28–37)^ To evaluate its potential as an IH lung cancer therapy, NanoPac suspension was administered to healthy rats through nebulized inhalation to determine the pharmacokinetic profile in both lung tissue and circulating plasma. Quantifiable levels of paclitaxel were detected in lung tissue at final necropsy, 2 weeks after single administration. In contrast, intravenous (IV) administration of nab-paclitaxel saw pulmonary paclitaxel concentrations fall below quantifiable levels after only 72 hours. Increasing body weights, clinical observations, and histopathological evaluation of lung tissue sampled at necropsy indicated an absence of treatment-related toxicity in all arms.^(25)^

In this study, we report the results of a preclinical pharmacology study using NanoPac delivered through nebulized inhalation to an orthotopic model of NSCLC in athymic nude rats.

## Materials and Methods

This study was conducted at Lovelace Biomedical (Albuquerque, NM); all animal and histopathological procedures were conducted under protocols approved by the Institutional Animal Care and Use Committee accredited by the Association for Assessments and Accreditation of Laboratory Animal Care International; immunohistochemistry (IHC) staining was performed at Reveal Biosciences (San Diego, CA) and Western Diagnostic Services Laboratory (Santa Maria, CA) using standard protocols.

### Animal model and cell culture

Male NIH-nru nude rats 3–5 weeks of age were used for the study (*n* = 127; Envigo, Greenville, IN). This nude rat strain has an autosomal recessive mutation on the rnu locus of chromosome 10, resulting in T cell deficiency, normal B cell counts, and increased numbers of NK cells and macrophages. The use of an athymic rodent was necessary to allow successful tumor engraftment in the model presented. Animals were quarantined for 2 weeks, randomized based on body weight, and whole body irradiated to induce further immunosuppression (∼500 rads, Phillips RT 250 X-ray Therapy Unit; Phillips Medical Systems, Shelton, CT) in preparation for Calu-3 NSCLC cell instillation.

Human lung adenocarcinoma cell line, Calu-3, was grown in Roswell Park Memorial Institute (RPMI) 1640 medium with 10% fetal bovine serum until 80% confluence at 37°C with 5% CO_2_ in cell culture flasks. Cells were harvested by trypsin dissociation then centrifuged at 100 *g* for 5 minutes; medium was removed, and cells resuspended to a concentration of 20 × 10^6^ cells in 450 μL of serum-free RPMI. Before instillation, 50 μL of 70 μM ethylenediaminetetraacetic acid (EDTA) was added to the cell suspension for a total intratracheal volume of 500 μL per rat. For instillation, animals were anesthetized by 3%–5% isoflurane in an induction chamber, gently secured, and Calu-3 cells were introduced into the lungs through the trachea. The animals underwent a tumor engraftment period of 3 weeks before initial treatment.

Water, lighting, humidity, and temperature control were monitored and maintained. Rats were fed standard rodent diet *ad libitum* during nonexposure hours.

### Chemicals, reagents, and suspension preparation

NanoPac and reconstitution solution were provided by CritiTech, Inc., (Lawrence, KS). The suspension for inhalation at 20 mg/mL concentration was prepared as described previously.^(25)^

Lovelace Biomedical obtained nab-paclitaxel (Abraxane^®^; Celgene Corporation, Summit, NJ) as clinical reference material. The drug product was reconstituted to 5.0 mg/mL with saline (Hospira, Lake Forest, IL) on the day of dosing and was stored as per manufacturer's instructions.

### NanoPac exposure system and conditioning

The NanoPac suspension was nebulized through two parallel Up-Mist (Hospitak, ConvaTec, McAllen, TX) compressed air jet nebulizers at a pressure of 20 psi. Aerosols were directed through a delivery line into a 32-port nose-only exposure chamber [depicted previously^(25)^]. Animals underwent exposure system conditioning over the 3 days before initial treatment; the first exposure lasting 30 minutes, the second lasting 60 minutes, and the third lasting 70 minutes. They were monitored closely throughout the conditioning periods and during exposures to ensure undue distress was not experienced.

### Treatment protocol and sampling

Methods for aerosol characterization, particle characterization, and deposited dose determination are detailed in a previous pharmacokinetic study^(25)^; pulmonary deposited doses were calculated based on weekly body weights and evaluated aerosol concentrations^(38)^; deposition fractions of 10% were used per Food and Drug Administration (FDA) recommendation for rodent inhalation of particles 1–5 μm.^(39,40)^ The results of the previous pharmacokinetic study were used to establish exposure durations of 33- and 65 minutes for 0.5 and 1.0 mg/kg doses in this study, respectively. Animals were randomized into six treatment groups (*n* = 20 each) with mean body weights ranging 254–260 g: no treatment (Group 1); IV nab-paclitaxel at 5.0 mg/kg once weekly for 3 weeks (Group 2); IH NanoPac at 0.5 or 1.0 mg/kg, once weekly for 4 weeks (Groups 3 and 4, respectively), or 0.5 or 1.0 mg/kg twice weekly for 4 weeks (Groups 5 and 6, respectively).

Animals were observed during drug administration, bodyweight sessions, and twice daily for signs of clinical changes, which were recorded as the primary measure of toxicity. Animal necropsy was scheduled 4 weeks after initial treatment; necropsy was performed by intraperitoneal overdose injection of a barbiturate-based sedative.

Left lung lobes were serially sectioned and allocated to histopathological or immunohistochemical analysis, fixed in formalin, and paraffin embedded. Right lung lobes were individually flash frozen.

### Histopathology

Lung tissue samples allocated to histopathology were trimmed at 4 μm, stained with Hematoxylin and Eosin (H&E), and shipped for external review to be graded subjectively and semiquantitatively by an experienced veterinary pathologist (Experimental Pathology Laboratories, NC). Observations in the lungs fell in two category grades; Adenocarcinoma and Tumor Regression. Grades were analyzed on a five-point scale: 0 indicating no involvement (0%); 1 indicating minimal involvement (∼1%–25%); 2 indicating mild involvement (∼26%–50%); 3 indicating moderate involvement (∼51%–75%); and 4 indicating marked involvement (∼76%–100%).^(41)^ Grades were recorded in an electronic pathology reporting system (PDS-Ascentos-1.2.0, V.1.2, NJ).

### Immunohistochemistry

A subset of 17 animals exhibiting the best response in each group (as determined by regression grading) were chosen for IHC evaluation (Group 1 *n* = 2; *n* = 3 each from Groups 2 to 6). Paraffin-embedded left lung blocks were sectioned at 4 μm thickness, collected on positively charged slides, and stained with H&E, Masson's Trichrome, anti-pan cytokeratin (anti-AE1/AE3), and anti-CD11b. A standard protocol was used for H&E and Masson's Trichrome staining. Review of immunohistochemical staining was completed independently from H&E sample review by an experienced clinical pathologist.

### Anti-pan cytokeratin (AE1/AE3) staining

Optimal keratin staining conditions were determined to be a 1:50 dilution of anti-pan cytokeratin antibody (anti-AE1/AE3, #ab27988, lot#GR3209978-1; Abcam) with heat-induced antigen retrieval using Leica Bond Epitope Retrieval Buffer 2 (EDTA solution, pH = 9.0) for 20 minutes. Nonspecific background was blocked with Rodent Block M (Biocare; #RBM961H, Lot #062117). Anti-AE1/AE3 antibody was detected using Mouse-on-Mouse HRP-Polymer (Biocare; #MM620H, Lot #062016) and visualized with 3′3-diaminobenzidine (DAB; brown). A Hematoxylin nuclear counterstain (blue) was applied. Mouse uterus was used alongside lung tissue samples as a positive control.

### Anti-CD11b staining

Optimal CD11b staining conditions were determined to be a 1:2000 dilution of rabbit anti-CD11b antibody (Abcam; #ab133357, lot#GR3209213-4) and heat-induced antigen retrieval using Leica Bond Epitope Retrieval Buffer 2 (EDTA solution, pH 9.0) for 20 minutes. Anti-CD11b antibody was detected using Novocastra Bond Refine Polymer Detection and visualized with DAB (brown). A Hematoxylin nuclear counterstain (blue) was applied. Rat lymph node tissue from a tissue bank was used as a positive control.

### BCL-6 staining

Anti-BCL6 antibody (Leica; Clone LN22) was used with heat-induced epitome retrieval using Leica Bond Epitope Retrieval Buffer 2 (EDTA solution, pH = 8.9–9.1) for 20 minutes. Nonspecific background was blocked with Peroxidase Block (HRP-Based Detection Kit). Anti-BCL6 antibody was detected and visualized using Leica Bond Polymer Refine Detection System. Bond polymer refine detection contained a peroxide block, post primary, polymer reagent, DAB chromogen and Hematoxylin counterstain, and supplied ready-to-use for the automated Bond System. Human tonsil tissue was used as a positive control.

### Statistics

*χ*^2^ group statistics were performed using Prism software (GraphPad, La Jolla, CA).

## Results

### Clinical observations, survival, and body weights

**FIG. 1.**
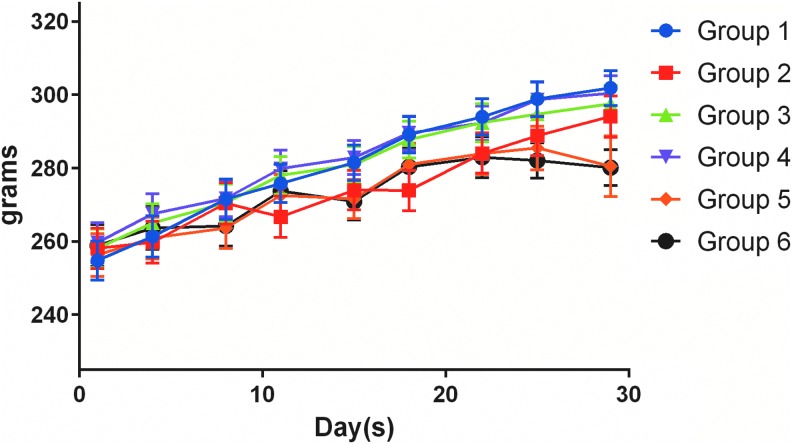
Average group body weights (grams) throughout the treatment period (±SEM). Group 1, untreated control; Group 2, 4.68 mg/kg intravenous nab-paclitaxel, once weekly for 3 weeks (per package insert); Group 3, 0.655 mg/kg inhaled NanoPac^®^, once weekly for 4 weeks; Group 4, 1.166 mg/kg inhaled NanoPac, once weekly for 4 weeks; Group 5, 0.64 mg/kg inhaled NanoPac, twice weekly for 4 weeks; Group 6, 1.176 mg/kg inhaled NanoPac, twice weekly for 4 weeks. SEM, standard error of the mean.

All animals gained weight at similar rates throughout the study (Fig. 1) and survived to designated necropsy. Observations related to the model noted in all treatment arms included skin rash and labored breathing. Tan nodules were noted on the lungs of all animals on study, including untreated controls during necropsy. Red and/or tan patchy discolorations of the lung were also noted during gross necropsy in all groups (Group 1 *n* = 4; Group 2 *n* = 1; Group 3 *n* = 8; Group 4 *n* = 5; Group 5 *n* = 1; Group 6 *n* = 5). A single abdominal hernia from an animal in Group 1, and a single nodule on the pericardium of an animal from Group 4 were also observed. No other abnormal gross observations were noted.

### Dose determination and aerosol characteristics

The average dose per administration for group 2 (IV nab-paclitaxel) was 4.68 mg/kg.

Droplet particle size distributions were measured weekly with an average of mass median aerodynamic diameter of 2.01 μm and average geometric standard deviation of 1.87 μm. The average Paclitaxel aerosol concentration inhalation exposure for once-weekly low dose and the once-weekly high dose was 270.51 and 244.82 μg/L, respectively. The paclitaxel average aerosol concentration for twice-weekly low dose and twice-weekly high dose was 263.56 and 245.76 μg/L, respectively. With their respective administration windows, the average achieved rodent deposited dose per administration for the once-weekly low dose, once-weekly high dose, twice-weekly low dose, and twice-weekly high dose were 0.655, 1.166, 0.640, and 1.176 mg/kg per inhalation, respectively.

### Histopathology

**FIG. 2.**
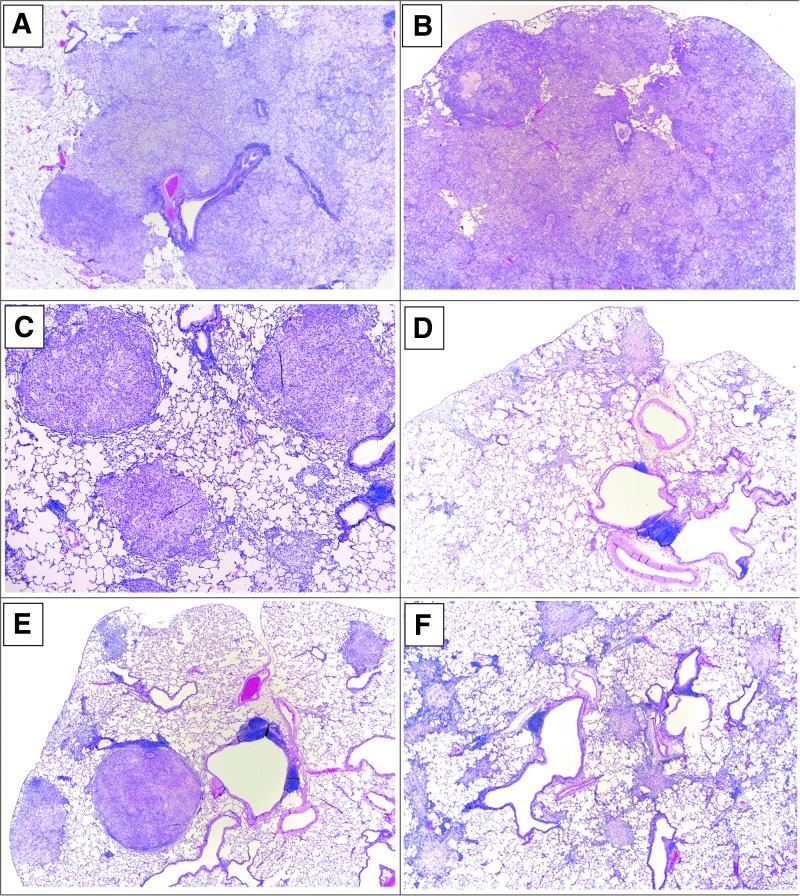
H&E photomicrographs. **(A)** Group 1: General distribution of undifferentiated, pleomorphic, large, anaplastic tumor cells within alveolar spaces or lining the alveolar septae. The majority of cells do not have features of adenocarcinoma and appear in sheets of contiguous tumor. Many cells have basophilic staining cytoplasm, while others are large, anaplastic and contain pale amphophilic staining. Note the presence of a preexisting resident population of alveolar macrophages. (**B)** Group 2: General distribution of large expansive tumor mass filling most alveolar spaces as well as neoplastic cells in the periphery. Most tumor cells are predominantly undifferentiated, pleomorphic, large, anaplastic with pale amphophilic staining. Immune cell infiltration are predominantly neutrophils and macrophages. **(C)** Group 2: Peripheral tumor masses with multiple smaller masses filling alveolar spaces. Tumor cells are pleomorphic, large, anaplastic with pale amphophilic staining. Tumor regression is present at the nodule peripheries with infiltration of macrophages. **(D)** Group 4: Previously populated tumor masses assessed by areas of residual fibrous connective tissue, central collagenous stroma and fibrocytes. Alveolar spaces are commonly filled with lymphocytic infiltrate. **(E)** Group 5: General distribution of regressing tumor masses. Regressing masses are variably small and randomly distributed. Fibrous connective tissue is seen filling/replacing alveolar spaces and suggests foci of regressing adenocarcinoma. Acute necrosis, fibrous connective scaffolding, mixed cell infiltration of macrophages, giant cells and lymphocytes in the epithelium, and stroma signifying tumor regression. **(F)** Group 6: Tumor regression is evidenced by previously populated tumor masses in multiple small areas of fibrous connective tissue replacing the alveolar spaces with a central collagenous stromal core, with thickening of the septae and fibrocytes infiltrating the alveolar spaces suggesting foci of previous adenocarcinoma cells. H&E, Hematoxylin and Eosin.

Histopathology was performed on H&E-stained lung tissue from all study animals; photomicrographs of left lung tissues are depicted in Figure 2. Lungs of the animals in all groups contained some evidence of tumor burden, characterized by the presence of expansile variably sized small masses randomly scattered within the lung parenchyma and larger masses that effaced >75% of the lung parenchyma, smaller airways, and blood vessels. A greater incidence of tumor regression was seen in NanoPac-treated versus untreated and nab-paclitaxel-treated animals evidenced by the presence of scalloping of the edges of the individual tumor masses as characterized by gradual to complete loss of tumor cells with residual fibrous connective tissue accompanied by infiltration of foamy macrophages (Table 1).

**Table 1.** Incidences and Severities of Tumor Regression as Assessed by Hematoxylin and Eosin Histopathology

	*Group*	*1*	*2*	*3*	*4*	*5*	*6*
Regression grade	None (0%)	20	18	9	9	9	7
	Minimal (∼1%–25%)		1	11	6	10	9
	Mild (∼26%–50%)		1		3		3
	Moderate (∼51%–75%)				1	1	
	Marked (∼76%–100%)				1		1
Total incidence			2	11	11	11	13

Following necropsy, left lung lobes were serially sectioned and fixed in formalin and paraffin embedded; regression was graded semiquantitatively. Incidence of regression was statistically higher in each IH NanoPac group as compared with either Group 1 or Group 2 (*p* < 0.01, *χ*^2^).

IH, inhaled.

### Histopathology—adenocarcinoma grade

The severity grade of adenocarcinoma included both undifferentiated and differentiated tumor nodules: undifferentiated adenocarcinoma morphology defined by lack of glandular formation, tumor cells with pleomorphic, large, anaplastic nuclei, and pale amphophilic staining with fine intracytoplasmic vacuoles resembling mucoid vesicles; differentiated adenocarcinoma defined as amphophilic stained tumor cells arranged in glandular patterns bound by alveolar septae.

H&E revealed tumor formation characterized by the presence of variably sized small masses randomly scattered within the pulmonary parenchyma, airways, and blood vessels by expanding and coalescing masses (Fig. 2). The larger masses were distributed primarily in the hilar regions or juxtaposed at the axial airway, whereas smaller masses were generally located in the peripheral lung fields. Adenocarcinoma was noted in most animals, confirming orthotopic tumor engraftment was successful in all groups; two IH NanoPac animal samples (Group 4 *n* = 1; Group 6 *n* = 1) had no evidence of residual tumor upon histopathological examination with signs of organizing stroma replacing areas presumed to have once contained tumor nodules.

### Histopathology—tumor regression grade

Tumor regression was identified by the scalloping of the edges of the individual tumor masses, characterized by irregular borders between tumor cell nodules and adjacent lung parenchyma with progressively smaller and more incohesive tumor cell collections resulting in the gradual to complete loss of tumor cells, with residual fibrous connective tissue scaffolding of the lung parenchyma accompanied by invasion of intervening stroma. Regression grades are summarized in Table 1. Unabated tumor growth was noted for all animals in Group 1. Although tumor regression was noted in all treatment arms, it was most common in IH NanoPac groups, with over half of the animals experiencing some degree of tumor regression in each group (*n* = 11–13/20 per group). Incidence of regression was statistically higher in each IH arm than either Group 1 or Group 2 (Table 1; *p* < 0.01, *χ*^2^).

### IHC subset

AE1/AE3^+^ staining resulted in sensitive and specific labeling with sharp demarcation between positively stained tumor cells and surrounding negatively stained non-neoplastic tissue. The CD11b^+^ staining of the IHC subset animals highlighted macrophages and NK cells; the staining was strong, sensitive, specific, and showed cytoplasmic membrane localization for both cell types. Grouped assessments are summarized in Table 2.

**Table 2.** Immunohistopathology Subset—Review of Regression, Immune Cell Infiltration, and Incidence of Lymphoid Structures

		*Regression*					*Immune cell infiltration*			
*Group number*	N	*0% of nodules*	*1%–10% of nodules*	*11%–50% of nodules*	*>50% of nodules*	*Complete regression*	*Mild*	*Moderate*	*Marked*	*TLS per low power*
1	2	2					2			0.5–1
2	3	1	1		1		1	2		1
3	3		1		2			1	2	3
4	3		1		1	1		2	1	2
5	3				3			2	1	2
6	3				2	1^a^		2	1	2

AE1/AE3^+^ staining highlighted tumor regression by the progressive loss of tumor cells at the periphery of tumor nodules resulting in irregular borders associated with increased immune cell infiltration, semiquantitatively graded as CD11b^+^ staining density: Mild (patchy distribution of scattered immune cells that are mostly single spaced); Moderate (increased density of immune cells that includes single-spaced individual cells and increased dense clusters of immune cells); and Marked (prominent and extensive immune cell infiltration that includes numerous dense collections of immune cells).

^a^ Residual keratin-positive structures were noted in one case; unable to distinguish between rare carcinoma cells or regenerative or atrophic entrapped blood vessels or alveoli. TLS per low-power field: 4 × objective and 10 × ocular.

TLS, tertiary lymphoid structures.

**FIG. 3.**
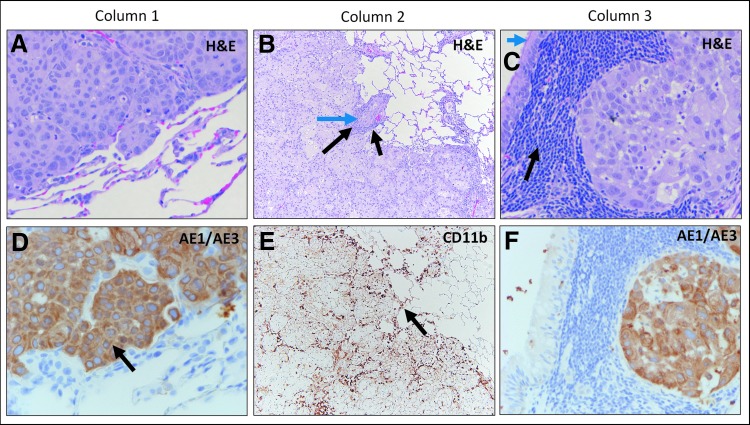
Group 1 H&E and IHC Staining. Column 1: H&E staining of adenocarcinoma **(A)** and corresponding keratin (AE1/AE3) staining **(D)** highlighting specific labeling of carcinoma cells (black arrow) in untreated lung tissue. Column 2: H&E staining of adenocarcinoma **(B)** showing focal rudimentary duct formation (blue arrow) with a limited immune cell component, consisting of lymphocytes and focal macrophages (black arrows). CD11b staining **(E)** highlighting minimal natural killer cells and macrophages (black arrow). Column 3: H&E staining of adenocarcinoma **(C)** next to bronchus-associated lymphoid tissue containing mature lymphocytes (black arrow). Note normal bronchial lining (blue arrow top left). Corresponding keratin (AE1/AE3) staining **(F)**, highlighting positively stained carcinoma cells and lack of lymphoid cell staining. IHC, immunohistochemistry.

Group 1 animals exhibited uniform growth of densely packed AE1/AE3^+^ adenocarcinoma cells with well-circumscribed and demarcated pushing margins from surrounding normal lung tissue (Fig. 3). Mild lymphoid and macrophage infiltrates were associated with the tumor nodules, which consisted primarily of small lymphocytes and lymphoid clusters of macrophages that were sparse and primarily located at the periphery of the tumor. The overall features were consistent with unabated tumor cell growth.

**FIG. 4.**
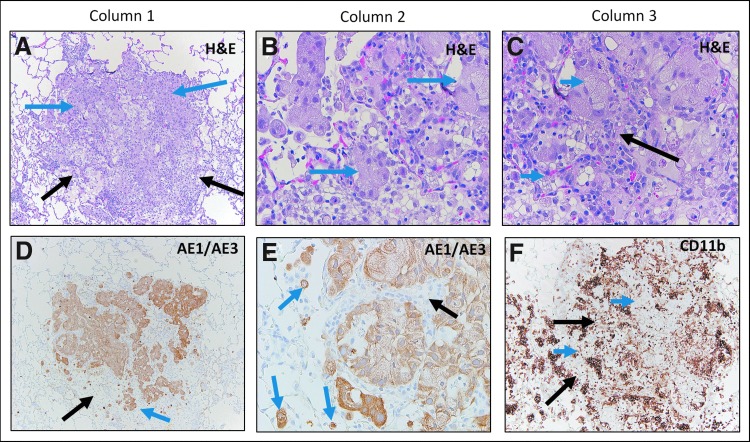
Group 2 H&E and IHC staining. Column 1: **(A)** H&E staining of adenocarcinoma (blue arrows) showing progressive separation of tumor cells and an increased immune cell response (black arrows). Corresponding keratin (AE1/AE3) staining **(D)** showing tumor cluster separation (blue arrow) and intervening stroma (black arrow). Column 2: Higher magnification view **(B)** of image **(A)**, showing smaller clusters of tumor cells (blue arrows) as compared with nontreated tissue. Keratin (AE1/AE3) staining **(E)** higher magnification view of image **(D)** showing separated tumor cell nodules, decreasing tumor cell clusters, and individual single tumor cells (blue arrows). Black arrow shows the unstained intervening stroma containing immune cells. Column 3: H&E staining **(C)** showing immune cells (black arrow) in center of tumor nodule (blue arrows). Corresponding low magnification view of a CD11b-stain **(F)** of image **(A)**, showing increased density of positively stained immune cells (black arrows) within the tumor cell clusters and residual carcinoma that is not labeled with CD11b (blue arrows).

Group 2 samples exhibited increased CD11b^+^ immune cell infiltration focally extending within the periphery of the AE1/AE3^+^ nodules in two of the three samples reviewed, highlighting separation of tumor cell clusters (Fig. 4). CD11b^+^ infiltrate between AE1/AE3^+^ nodules suggest partial tumor regression in comparison to the untreated control group.

**FIG. 5.**
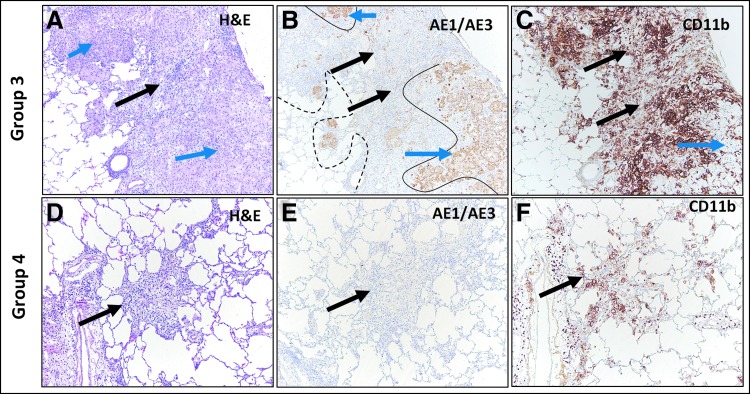
Group 3 and 4 H&E and IHC staining. Group 3: H&E staining of adenocarcinoma **(A)** showing regression highlighted by separation and loss of tumor cells at tumor periphery (blue arrows) and non-neoplastic stroma with inflammation separating the carcinoma into nodules (black arrow). Keratin (AE1/AE3) staining **(B)** showing positively stained residual carcinoma (blue arrows), original carcinoma border (dashed black line) and residual carcinoma border (continuous black lines). Unstained area (black arrows) represents a large area of tumor loss. CD11b staining **(C)** showing positively stained immune cell infiltration in areas of tumor regression (black arrows). Residual carcinoma is unstained (blue arrow). Group 4: H&E staining **(D)** showing no viable adenocarcinoma: complete regression, with organizing inflammation composed of fibrous stroma with admixed lymphocytes and macrophages (black arrow). Lack of positive keratin (AE1/AE3) staining **(E)** in the same area as image **(D)** confirms morphologic absence of residual carcinoma (black arrow). CD11b stain **(F)** showing mild-to-moderate immune cell infiltrate (black arrow).

**FIG. 6.**
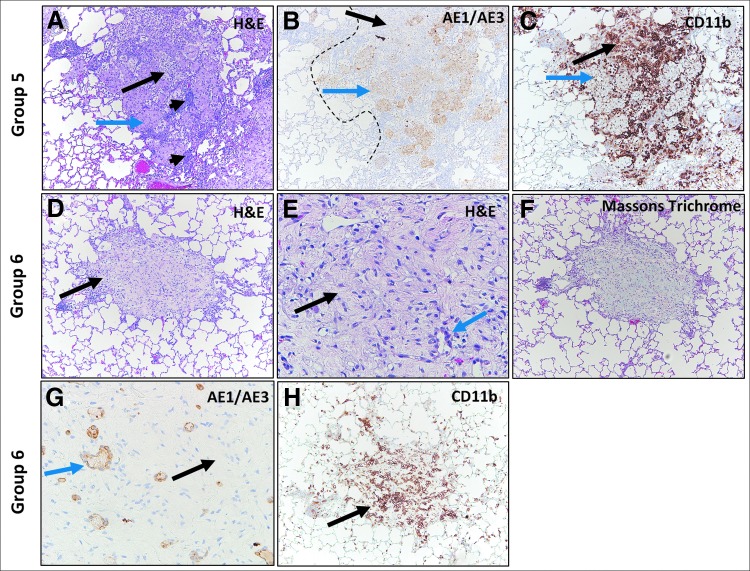
Group 5 and 6 H&E and IHC Staining. Group 5: H&E staining of adenocarcinoma **(A)** showing regression within a tumor nodule (blue arrow), increased intranodular stroma (long black arrow) and increased intra- and perinodular lymphoid cells (short black arrows). Keratin (AE1/AE3) staining **(B)** showing positively stained carcinoma (blue arrow) and large unstained area of tumor loss (black arrow); original carcinoma border (dashed black line). CD11b staining **(C)** shows positively stained immune cell infiltrate in area of regression (black arrow) and surrounding areas of unstained carcinoma (blue arrow). Group 6: H&E staining showing cluster of foamy cells (black arrow) **(D)**. Increased magnification **(E)** of image **(D)** shows cells with foamy cytoplasm (black arrow) and duct-like structures or regenerating small blood vessels or alveoli (blue arrow). Masson Trichrome stain **(F)** showing blue-stained collagen (fibrous organization). Keratin (AE1/AE3) staining **(G)** labeling duct-like structures (blue arrow) that may represent a combination of regenerative or atrophic non-neoplastic structures such as small blood vessels or alveoli; carcinoma could not be definitively excluded. Note unstained intervening foamy cells (black arrow). CD11b staining **(H)** corresponding to image **(D)**, shows positively stained immune cell infiltrate in area of tumor regression (black arrow).

IH NanoPac groups exhibited features consistent with moderate to marked regression in all subset samples reviewed. Marked intratumoral lymphoid infiltrate supports increased immunosurveillance, and tumor cell recognition and clearance in comparison to Groups 1 and 2. Representative photomicrographs of tumor regression and clearance in IH NanoPac groups are displayed in Figure 5 and 6.

Lung tissue samples from two animals (Group 4 *n* = 1; Group 6 *n* = 1) exhibited features of pathological complete response; no evidence of viable tumor with multiple foci of organizing tissue and immune cells (Figs. 5 and 6); Masson's Trichrome staining these foci blue, consistent with collagen deposition (Fig. 6). AE1/AE3^+^ stained focal keratin-positive cells and small duct-like structures within these foci; it could not be determined whether these positively stained cells represented residual tumor cells, small admixed regenerative blood vessels with keratin-labeling of the endothelial lining, or reactive, regenerative, and atrophic non-neoplastic lung parenchyma (Fig. 6). While immunohistochemical analysis was limited to 17 animals out of a total 120 animals evaluated histopathologically, the results reinforce the H&E review findings.

H&E and AE1/AE3 analysis revealed small dense collections of lymphoid cells and lymphoid structures (LSs) in all lung samples (commonly referred to as bronchus-associated lymphoid tissue^42^). In the two Group 1 IHC samples, the occurrence of LSs were generally sparse (0.5–1 LS per low-power field) and composed of dense collections of small mature lymphocytes with inconspicuous nucleoli.

**FIG. 7.**
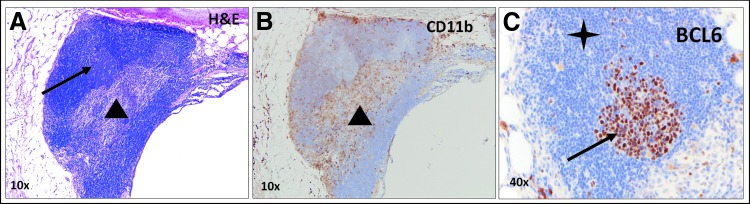
Tertiary lymphoid structures. **(A)** H&E staining showing a tertiary lymphoid structure and adjacent bronchus lumen. The tertiary lymphoid structure has an organoid appearance with lymphoid follicles composed of mantle zone B cells surrounding germinal centers composed of B cells (black arrow), as well as a sinus containing histiocytes and lymphocytes (black triangle). **(B)** CD11b staining showing macrophages within the sinus (black triangle). **(C)** BCL-6 staining shows positively staining follicle center-type B cells localized within the germinal center (black arrow) and unstained surrounding mantle zone B cells (black star).

In contrast to control animals, treatment group samples exhibited germinal center formation with the greatest density noted in Group 3 (*n* = 3/low-power field). The structures were composed of well-circumscribed collections of dense lymphoid tissue with varying degrees of architectural maturation that included lymphoid follicles, intrafollicular areas, and paracortical zones. The smaller collections of lymphoid cells associated with the tumors were composed predominantly of compact, mature-appearing lymphocytes that ranged in size, appearing both at the periphery and focally within tumor nodules. To further characterize these structures, a single slide from an animal in Group 6 was stained with BCL-6 for B cell identification, which appeared to stain an active germinal center within one of these structures (Fig. 7). Due to the proximity of these LSs adjacent to tumor nodules exhibiting features of regression, a local adaptive immune response may be occurring through tumor-associated antigen presentation.

## Discussion

Although paclitaxel has been approved by the FDA as an IV NSCLC treatment for over 15 years, the response rate to IV paclitaxel averages only 30%–40%.^(43)^ With a relatively high molecular weight and limited aqueous solubility, paclitaxel may have limited pulmonary exposure due to poor absorption from the peripheral blood following IV administration.^(44)^ As the efficacy of paclitaxel in the treatment of NSCLC is correlated with its concentration,^(45)^ alternate routes of administration have been sought to increase both locoregional dose and retention.^(22)^ These include direct inhalation of a cytotoxic in a variety of formulations, including colloidal dispersions, microparticles and nanoparticles of various polymers, liposomes, and other lipid-based formulations.^(46)^ While no IH therapies have been approved for the treatment of NSCLC, clinical trials have reported benefits following IH granulocyte/macrophage colony-stimulating factor, IL-2, as well as cytotoxic treatments, including doxorubicin, gemcitabine, carboplatin, and cisplatin.^(47–59)^

This study described the multiadministration treatment effect of IH NanoPac in an orthotopic athymic rat model of NSCLC. The reconstituted suspension was successfully nebulized on all occasions and measured for dosing without issue; average doses per administration were assessed to be greater than those desired, but <0.18 mg/kg above target values. Repeat administration of drug was handled well in all groups compared with Group 1; no discernable difference in body weight throughout the study, and no in-life observations of note were reported. IH NanoPac groups demonstrated a treatment benefit evidenced by increased tumor regression, occasional complete tumor eradication, and accompanying tumor nodule separation through increased density of infiltrating stroma. Taken together, these observations suggest that the response of the tumor to prolonged exposure of tumoricidal concentrations of paclitaxel may involve two different mechanisms of action: (1) direct tumoricidal effect resulting in the production of neoantigens and antigen spread stimulating the immunogenic tumor cell death cycle to further the tumoricidal response to therapy, and (2) the immunostimulatory effect of paclitaxel, including infiltration of macrophages, B lymphocytes, NK, and dendritic cells.

The immune cell infiltration of lymphocytes and macrophages within tumor nodules may provide patients with NSCLC further benefit by priming tumor cell recognition ahead of IV checkpoint inhibitors' administration.^(3,5,8–10,60)^ The presence of tertiary LSs (often referred to as bronchus-associated LSs in the lung^42^) located within and around tumor nodules following inhalation treatment was noted, which has been previously reported to be associated with better outcomes in patients with NSCLC.^(61)^ Similar observations were reported by Yuen et al. in that the number of LSs, particularly containing B cells, correlated directly with positive outcomes in both human disease and mouse models.^(61–64)^ BCL-6 staining showed lymphatic activity by the presence of B cells at the center of the LSs, consistent with active lymphoid follicles.

While the immunostimulatory versus cytotoxic effects cannot be distinguished from one another in the orthotopic athymic model presented, the infiltrating stroma can lead one to theorize greater efficacy may be experienced in an immunocompetent model with an active immunogenic tumor cell death cycle. These results encourage further development of IH NanoPac as an IH therapy for lung cancer; dual-species repeat-dose good laboratory practice (GLP) toxicology studies are underway to further characterize the safety and tolerability profile and to qualify first in-human clinical trials.

## Notes

Reviewed by:

Remi Rosiere

Zimeng Wang
